# Foot typology, body weight distribution, and postural stability of adolescent elite soccer players: A 3-year longitudinal study

**DOI:** 10.1371/journal.pone.0204578

**Published:** 2018-09-28

**Authors:** Jitka Marencakova, Tomas Maly, Dai Sugimoto, Tomas Gryc, Frantisek Zahalka

**Affiliations:** 1 Sport Research Center, Faculty of Physical Education and Sport, Charles University, Prague, Czech Republic; 2 Orthopedic Center, Children’s Hospital Boston, Boston, United States of America; 3 The Micheli Center for Sports Injury Prevention, Waltham, United States of America; 4 Department of Orthopaedic Surgery, Harvard Medical School, Boston, United States of America; University of Illinois at Urbana-Champaign, UNITED STATES

## Abstract

**Objective:**

The unique foot morphology and distinctive functions facilitate complex tasks and strategies such as standing, walking, and running. In those weight-bearing activities, postural stability (PS) plays an important role. Correlations among foot type, PS, and other musculoskeletal problems that increase sport injury risk are known. However, long-term associations among the foot type, the PS, and body weight (BW) distribution are lacking. Thus, the purpose of this study was to longitudinally identify changes in foot morphology, PS, and symmetry in BW distribution during adolescence among elite male soccer players.

**Methods:**

Thirty-five Czech elite male soccer players (age, 15.49 ± 0.61 years; BW, 64.11 ± 6.16 kg; body height, 174.62 ± 5.71 cm) underwent foot type, PS, and BW distribution measurements during 3 consecutive years (T1, T2, T3). The Chippaux-Smirak index (CSI), BW distribution, and centre of pressure (COP) displacement (total traveled way [TTW]) of each player for the preferred (PL) and non-preferred leg (NL) were acquired. Repeated-measures analysis of variance (RM ANOVA), Bonferroni´s post hoc tests, and partial eta-squared (η_p_^2^) coefficient were used for investigating the effect of time on selected variables and effect size evaluation.

**Results:**

Statistically significant effect of time on CSI values (PL: F_2,68_ = 5.08, p < 0.01, η_p_^2^ = 0.13 and NL: F_2,68_ = 10.87, p < 0.01, η_p_^2^ = 0.24) and COP displacement values (PL: F_2,68_ = 5.07, p <0.01, η_p_^2^ = 0.13; NL: F_2,68_ = 3.53, p <0.05, η_p_^2^ = 0.09) for both legs over 3-years period was identified. Furthermore, the Bonferroni´s post hoc analysis revealed a significant improvement of PS values in the PL (TTW_T1_ = 1617.11 ± 520.22 mm vs. TTW_T2_ = 1405.29 ± 462.76, p < 0.05; and between TTW_T1_ = 1617.11 ± 520.22 mm vs. TTW_T3_ = 1370.46 ± 373.94, p < 0.05). Only BW distribution parameter showed no significant differences, although slightly improved over time.

**Conclusions:**

We observed changes in foot typology, PS, and BW distribution in young elite male soccer players during 3 consecutive years. Results demonstrated that changes in PS and body weight distribution under the high-load sport conditions during adolescence may improve with aging, except for foot morphology. Therefore, foot morphology should be carefully monitored to minimize sport injury risk in professional young soccer players during adolescence. Further research is necessary to determine more clear associations between these parameters, soccer-related injuries, and sport performances.

## Introduction

The foot is a unique anatomic, neurophysiologic, and functional structure, which facilitate complex tasks and strategies such as standing, walking, and running in human movements [1]. The foot also plays a vital role for maintaining the postural stability (PS) during these movements [2]. Controlling PS requires enough flexibility to absorb shock and adapt to an uneven terrain and, at a different movement phase, conversely sufficient rigidity to create an impulse to move forward [3]. Thus, a certain structural and functional alignment simply described as a foot type (FT) influences foot and lower limb functions, and kinematic gait parameters [4–8], and leads to PS difficulties and musculoskeletal disorders [9]. Current evidence shows that flexible flatfoot type and its poor function are risk factors of sport injuries [10–12].

Soccer players are exposed to high risks of musculoskeletal injuries. In professional soccer, the incidence of injury is 8 cases per 1000 hours [13]. Previous studies reported that the most frequent injury in soccer is caused by contact with an opponent [14], whereas current research showed an increase for non-contact mechanisms of injuries, such as ball taking, jumping, running, change of direction speed, shooting and landing [15]. These tasks require having a good PS control. Lower balance ability has been reported to be significantly associated with an increased risk for ankle injuries in male adult soccer players [16]. Furthermore, another study indicated a direct correlation between flatfoot type and postural control. This study identified that the worse the flatfoot arch is, the worse the postural control is [17] in a meaning of a poor foot alignment. Some studies mentioned that asymmetry of body weight (BW) distribution between the feet leads to overuse of one leg [18]; and according to a review study that focused on soccer injuries reported, up to 24% of all soccer injuries are classified under overuse type [19]. Soccer players usually use one leg frequently for kicking, passing, shooting, and leading the ball. Long-term participation in these single leg activities may lead to overload of one side of the body (legs); thus, muscle imbalances or asymmetries may appear unless they are adequately compensated [20]. Bilateral comparison of the muscle strength in the lower limbs (bilateral ratio; preferred vs. non-preferred leg) or comparison between agonist and antagonist strengths (ipsilateral ratio; e.g., knee flexor vs. knee extensor) may indicate potential weaknesses of the muscle system that are predisposed to muscle injury and can negatively influence sport performance [21–24]. In addition to the asymmetry, growth/maturation factors may play an important role. For example, several investigations reported that adolescents have either the same or more frequent injury occurrence than adult football players [25]. This may potentially stem from growth spurt factor during adolescence. Additionally, a repetitive long-term high load acting during this critical part of aging may have an influence on the performance. Moreover, this may further negatively impact one’s health status in their adulthood. Recently, a few studies were conducted using a longitudinal study design in youth soccer players, especially the injury incidence [26], a relationship of peak high velocity and performance [27], and, also psychological factors in adolescent soccer players [28]. However, no study had investigated the long-term progression of such specific factors as foot morphology, BW distribution, and PS in young professional soccer players.

Hence, the aim of our longitudinal study was to register longitudinal changes in foot typology, PS, and symmetry in BW distribution among adolescent elite soccer players during aging to adulthood within 3 consecutive years.

## Materials and methods

### Study design

A prospective, longitudinal study design was used to attain the purpose this study. The research was approved by the ethics committee of the Faculty of Physical Education and Sports at Charles University in Prague prior to commencement of this project. Each participant or his legal guardian was fully informed about the study design and signed an informed consent form.

### Subjects

Seventy-five elite male soccer players from the national teams of Czech Republic initially agreed to voluntary participate in the study. However, in the first year, we lost 13 players (N = 62). In the second year, we further lost 13 players (N = 49), and during the third year, additional 14 players did not participate in this study (N = 35). Thus, at the end only 35 players completed the 3-year longitudinal study (35 men; age, 15.49 ± 0.61 years; BW, 64.11 ± 6.16 kg; and body height, 174.62 ± 5.71 cm) and each lower limb of participant was investigated separately. The reason of dropout was mostly because of the exclusion criteria including lower limb, pelvic, or spinal injury within 6 months before the study started or at any time during the study period, and any acute musculoskeletal pain. Also, several players changed their soccer team affiliations during the study period or relocated to another geographic region. All data were collected one time per a year for 3 consecutive years. The timing of data collection was always during a pre-season in each year. Thus, each player followed measurements three times in total (T1, T2, T3). Measurements were conducted in accordance with the ethical standards of the Declaration of Helsinki and sport and exercise science research [29].

### Measurements and procedure

The laboratory protocol for the methodology for postural stability, body weight distribution, and foot typology assessment is described in full at http://dx.doi.org/10.17504/protocols.io.QP8DVRW

#### Assessment of postural stability and body weight distribution

This prospective, a longitudinal study of foot morphology, symmetry of BW distribution, and PS characteristics of elite soccer players was performed using the RS Footscan Balance 7.6 second-generation tensometric plate (RSscan International, Belgium). The PS and BW distribution between the lower limbs during quiet standing were tested using the Footscan plate with a dimension of 50 x 40 cm and 4 100 sensors with a precision of 0.1 N.cm^−2^, a sampling rate of 10 Hz. The measuring plate was placed 2 m from the wall, where a visible point was placed exactly in the perpendicular vertical axis of the body of each player standing barefoot on the plate and at the level of the height of the eyes.

For analysis of PS, we chose the centre of pressure (COP) displacement parameter expressed as total traveled way (TTW) in millimetres. We used the standard procedure described in previous studies [30]. Firstly, the calm narrow standing (NS) test was performed, and then in two single-leg tests, PS was measured during preferred leg (PL) and non-preferred leg (NL) standing. Players’ PL was identified as their preferred leg when performing soccer tasks, such as kicking, passing, tackling, and dribbling.

The NS test lasted for 30 s, and the one-leg stance tests took 60 s, with a 60-s rest between tests. In a standard protocol, a researcher informed the player when each test started and finished. In the beginning of NS test, the player stood in the middle of the measuring plate, with his feet as close as possible, but not touching each other, and arms alongside the body while he was instructed watching the point directly and being motionless during the testing. Then, the researcher started the 30s data collection. After finishing the test and 60s resting, the one-leg stance test was conducted for each leg. Before the test had started, the researcher instructed the player to watch the point and be motionless during the one-leg test. Then, the player could choose the leg he wanted to start with, and he stood on that leg. After that, he bended the non-stance leg knee up to 90° of flexion with a concurrent neutral position in the hip joint and held arms alongside the body. When the preparation was completed, the 60s one-leg stance test was conducted. After the first test, a 60s rest and a new process of the preparation followed for the second test. The player changed his legs and repeated the equal one-leg stance test with the same instructions but for the second leg.

From the NS test, we found the percentage values of BW distribution between the legs (BWD_Δ_) and set the normative value of asymmetry for the 5% difference [31]. The TTW parameter of one-leg standing was detected for each leg. The players followed 3 trials for each standing test with open eyes, and the mean values were calculated.

#### Assessment of foot typology

For measurements and calculation of the Chippaux-Smirak index (CSI), which is a validated index for foot typology description [32], we used one-leg standing footprints to evaluate the foot type of each lower limb separately. CSI is a ratio of the minimum width of the midfoot to the maximum width of the forefoot [33], with good intra- and inter-rater reliability [32]. The classification scale of the CSI describes 5 categories of medial longitudinal foot arch as follows: height arch (< 0%); normal arch (N; 0.1–29.9%); intermediate foot arch (I; 30.0–39.9%); and lowered foot arch (L; 40.0–44.9%) and flatfoot (F; > 45.0%) [34]. According to other authors, we can further divide flatfoot type by the degree of its severity as follows: mild (45.1–50.0%), moderate (50.1–60.0%), and severe (6.1–100%) [35].

### Statistical analysis

For statistical processing of the data, we used descriptive and inductive statistics. Measurements were expressed using the arithmetic mean, and the measure of variability was expressed using standard deviation. Data normality was evaluated using the Shapiro-Wilk test. To assess the significance of the independent variables (time of observation) based on the dependent variables a repeated-measures analysis of variance (RM ANOVA) was used. To evaluate sphericity, an assumption of RM ANOVA, the Mauchly test was performed. Multiple comparisons of the means of the monitored variables were performed using the Bonferroni´s post hoc test. Moreover, the effect size coefficient was assessed using partial eta squared (η_p_^2^). The foot type differences over three observed years were analysed by non-parametric χ^2^ (chi square test) and Cramer’s V. For all the analyses, the statistical significance level was set as a p value of 0.05 to reject the null hypotheses. Statistical analysis was performed using IBM SPSS v21 (Statistical Package for Social Sciences, Inc., Chicago, IL, 2012).

## Results

### Physical characteristics and body weight distribution

Thirty-five elite football players completed the study. The basic characteristics of the participants and their BW distribution in 3 consecutive years are shown in Table 1. The mean BW distribution was more concentrated in the NL than PL throughout the 3-year period. According to narrow standing test the mean BW distribution asymmetry ranged from 12.2% to 15.2% and had a slightly, but not significant declining trend on time progression.

**Table 1:** Characteristics of participants and body weight distribution in 3 consecutive years (T1, T2, T3).

Parameters		Descriptive statistics				RM ANOVA			Bonferroni’s post-hoc test (p < 0.05)
		Mean	SD	95% CI interval		F	p	η_p_^2^	
				Lower	Upper				
Body height (cm)	T1	174.63	5.71	172.67	176.59	70.79	<0.01	0.68	T1 vs. T2T1 vs. T3T2 vs. T3
	T2	175.90	5.74	173.93	177.87				
	T3	177.94	5.45	176.07	179.81				
Body mass (kg)	T1	64.11	6.17	62.01	66.23	76.32	<0.01	0.69	T1 vs. T2T1 vs. T3T2 vs. T3
	T2	65.95	6.17	63.84	68.07				
	T3	70.09	6.45	67.89	72.31				
Foot size (UK)	T1	8.63	1.03	8.23	8.98	11.68	<0.01	0.26	T1 vs. T2T1 vs. T3
	T2	8.87	1.00	8.53	9.22				
	T3	8.91	1.00	8.57	9.26				
BWD_PL_ (%)	T1	46.91	8.71	43.92	49.91	0.03	>0.05	0.00	
	T2	47.11	11.11	43.30	50.93				
	T3	46.69	7.49	44.11	49.26				
BWD_NL_ (%)	T1	53.23	8.71	50.23	56.22	0.19	>0.05	0.00	
	T2	52.86	11.13	49.04	56.68				
	T3	53.10	7.58	50.50	55.70				
BWD_Δ_ (%)	T1	13.51	12.50	9.22	17.81	0.53	>0.05	0.02	
	T2	15.17	17.07	9.31	21.04				
	T3	12.19	10.79	8.48	15.89				

BWD_PL_, body weight distribution preferred leg; BWD_NL_, body weight distribution non-preferred leg; BWD_Δ_, body weight distribution difference between legs; SD, standard deviation; CI, confidence interval.

### Postural stability

The COP displacement values of the group at time T1, T2, and T3 are depicted in Table 2. COP displacement expressed as TTW in millimetres had a significant decreasing tendency over 3 years for both legs (PL: F_2,68_ = 5.07, p < 0.01, η_p_^2^ = 0.13; NL: F_2,68_ = 3.54, p = 0.04, η_p_^2^ = 0.09). The Bonferroni post hoc analysis revealed a significantly higher level of PS in favour of the observed time in PL (TTW_T1_ = 1617.11 ± 520.22 mm vs. TTW_T2_ = 1405.29 ± 462.76, p = 0.02 and TTW_T1_ = 1617.11 ± 520.22 mm vs. TTW_T3_ = 1370.46 ± 373.94, p < 0.05).

**Table 2:** Centre of reassure displacement time dependence.

Parameters		Descriptive statistics				RM ANOVA			Bonferroni’s post-hoc test (p < 0.05)
		Mean	SD	95% CI interval		F	p	η_p_^2^	
				Lower	Upper				
**TTW**_**PL**_ **(mm)**	T1	1617.11	520.22	1438.41	1795.82	5.07	< 0.01	0.13	T1 vs. T2T1 vs. T3
	T2	1405.29	462.76	1246.32	1564.25				
	T3	1370.46	373.94	1242.01	1298.91				
**TTW**_**NL**_ **(mm)**	T1	1661.31	460.07	1503.27	1819.36	3.54	<0.05	0.09	T1 vs. T3
	T2	1562.31	523.55	1382.47	1742.16				
	T3	1414.69	534.69	1231.01	1598.36				
**TTW**_**Δ**_ **(mm)**	T1	333.69	229.71	254.78	412.59	3.34	<0.05	0.09	T1 vs. T3
	T2	283.03	287.14	184.39	381.66				
	T3	196.29	172.63	136.99	255.59				

TTW_PL_, total travel way preferred leg; TTW_NL_, total travel way non-preferred leg; TTW_Δ_, total travel way difference; SD, standard deviation; CI, confidence interval; T1, T2, T3 time of measurement.

### Foot typology

Repeated measurement ANOVA revealed a significant effect of the main factor (Time) on the CSI values for both legs (PL: F_2,68_ = 5.08, p < 0.01, η_p_^2^ = 0.13 and NL: F_2,68_ = 10.87, p < 0.01, η_p_^2^ = 0.24) (Table 3). The participants presented slightly higher CSI values of the PL (CSI_PL_) during the whole period in comparison with the NL (CSI_NL_). Significant differences were found between the CSI_PL_ values in years T2 (38.99 ± 9.10%) and T3 (42.06 ± 8.75%; p < 0.05) and CSI_NL_ values between years T1 (38.01 ± 7.85%) and T3 (41.91 ± 8.98%; p < 0.01), and between T2 (37.04 ± 9.36%) and T3 (41.91 ± 8.98%; p < 0.01).

**Table 3:** Chippaux-Smirak index time dependence.

Parameters		Descriptive statistics				RM ANOVA			Bonferroni’s post-hoc test (p < 0.05)
		Mean	SD	95% CI interval		F	p	η_p_^2^	
				Lower	Upper				
**CSI**_**PL**_ **(%)**	T1	39.50	8.35	36.63	42.37	5.08	< 0.01	0.13	T2 vs. T3
	T2	38.99	9.10	35.86	42.11				
	T3	42.06	8.75	39.05	45.06				
**CSI**_**NL**_ **(%)**	T1	38.01	7.85	35.31	40.70	10.87	< 0.01	0.24	T1 vs. T3T2 vs. T3
	T2	37.04	9.36	33.82	40.25				
	T3	41.91	8.98	38.82	44.99				

CSI_PL_, Chippaux–Smirak index preferred leg; CSI_NL_, Chippaux–Smirak index non-preferred leg; SD, standard deviation; CI, confidence interval; T1, T2, T3 time of measurement.

**Fig 1:**
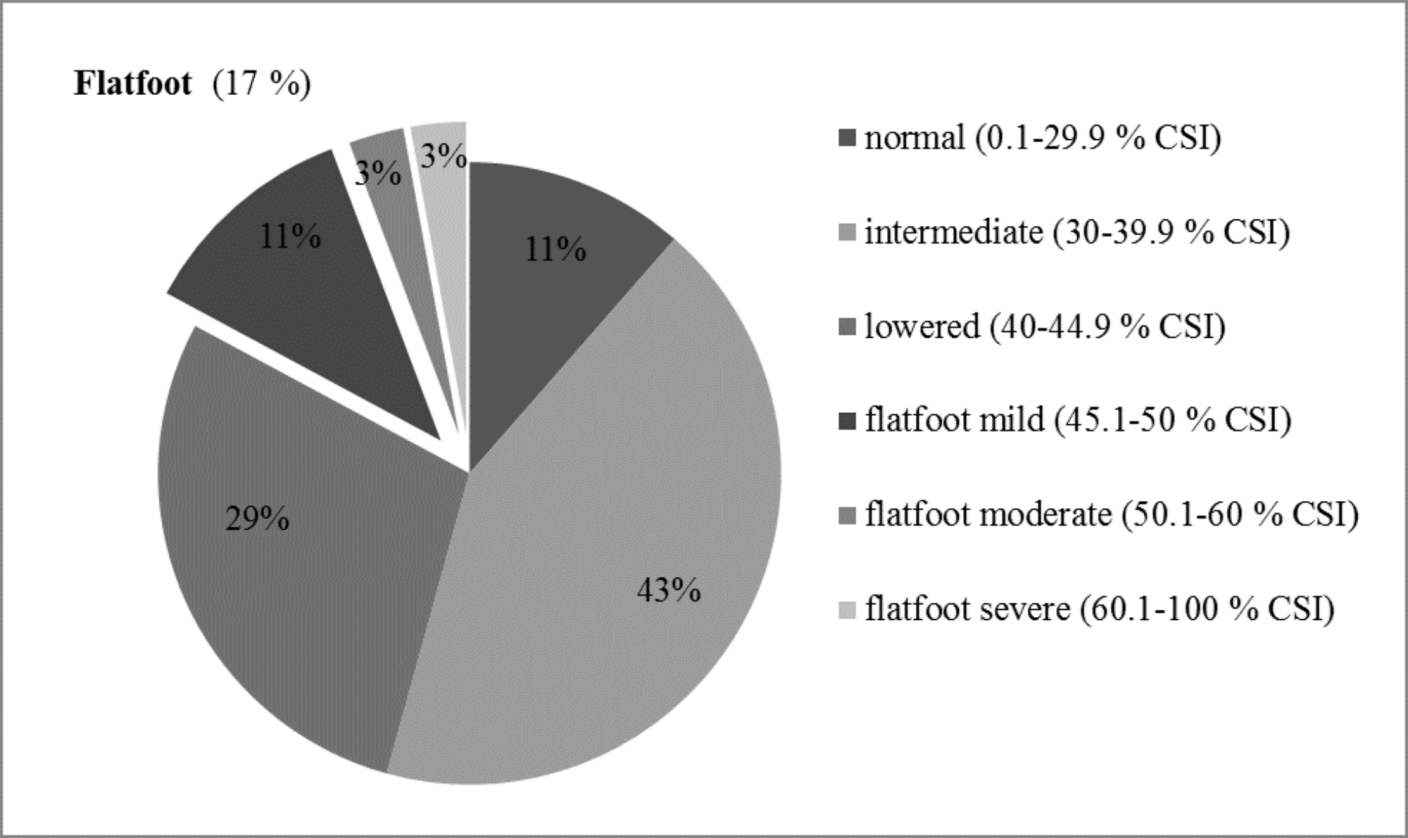
Foot type distribution according to CSI in time T1 (n = 70).

**Fig 2:**
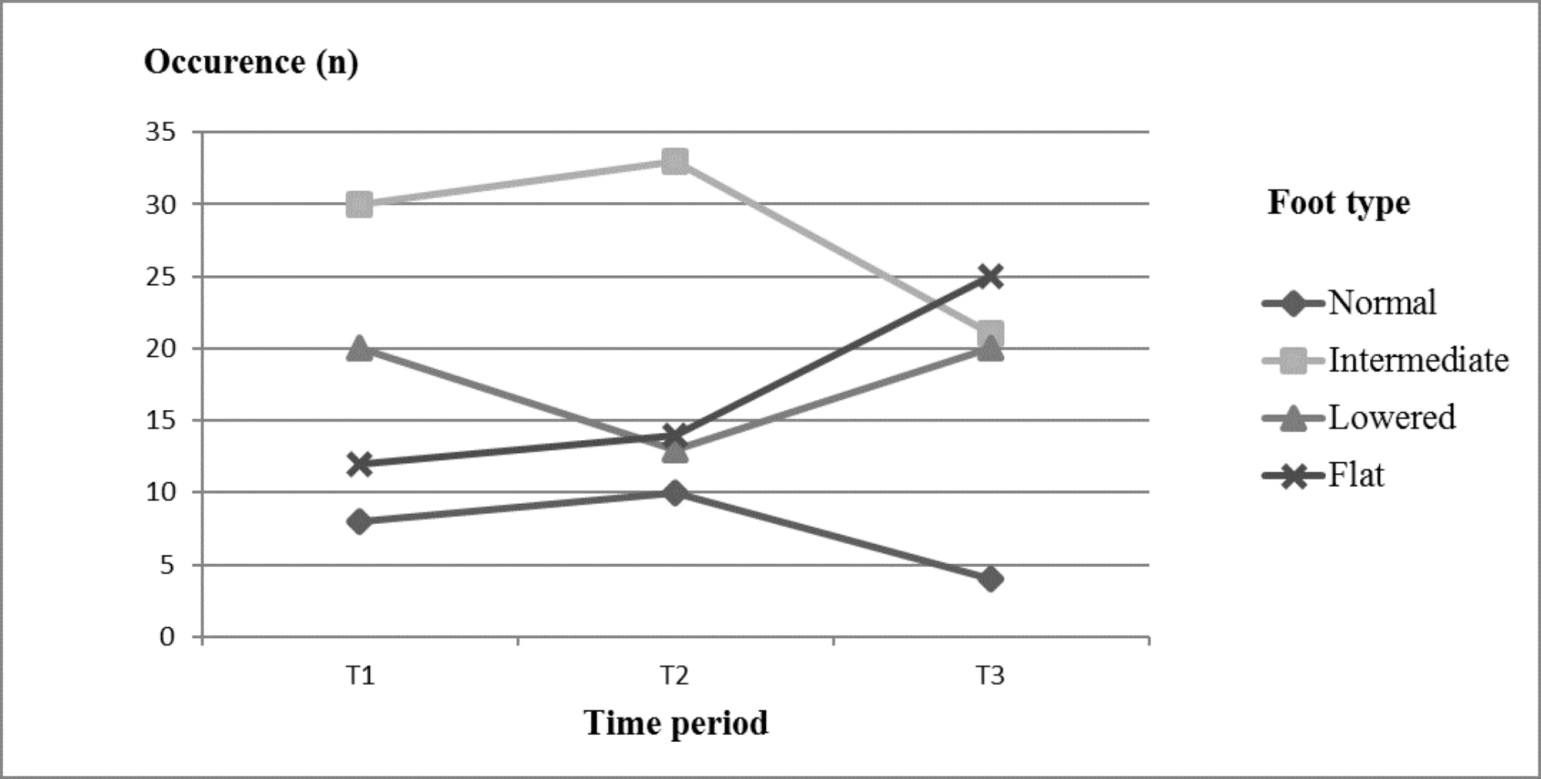
Time dependent progression of foot type distribution (n = 70).

Foot types distribution and time progression are shown in Figs 1 and 2. The foot types differences over three observed years were significant (χ = 12.94, p < 0.05, Cramer’s V = 0.04). According to CSI, the most frequent FT at T1 was intermediate foot arch (I_T1_ = 43%), followed by the lowered (L_T1_ = 29%) and flatfoot (F_T1_ = 17%) types. Only 11% of the participants had a normal arch foot type. Intermediate and normal FTs had slightly increasing level of distribution at T2 but showed a prominent decreasing tendency at T3 in the sample representation. On the other hand, the lowered FT initially showed a decreasing trend in the distribution but, at T3, increased back to the same value as at T1. Only the flatfoot type had a purely increasing tendency in the sample representation throughout the whole 3-year period (F_T2_ = 20%; F_T3_ = 36%).

## Discussion

The purpose of this study was to observe progression in foot morphology, BW distribution symmetry, and PS changes during the adolescence period of elite male soccer players. In our research sample, we found that the BW distribution asymmetry in between legs became higher than the previously reported value of 5% [31]. Concretely, it ranged from 12.2% to 15.2%. However, no statistically significant changes were found during the period; only a slightly decreasing tendency with aging was depicted. In previous studies, this topic was mostly connected with neurological diseases such as hemiplegic stroke, in which patients show more severe BW distribution asymmetry, and it was identified as a significant risk factor of falling. One study showed that stroke patients had a mean value of BW distribution asymmetry of approximately 49%, which declined to approximately 38%, with declining incidents of falls, after a special training [36]. Unlike studies that dealt with stroke patients, in a field of sports medicine and exercise science, there has not been a report of BW distribution analysis based on asymmetry, as we know recently; thus, this study is novel. The only one study that is linked with the current result, we found, was a study performed by Junge and Dvorak [19]. According to their review, the BW asymmetry had a considerable impact on overuse injury. The asymmetry observed in this study was probably caused by lateral dominance of the lower limbs, which may stem from unilateral sports such as soccer. In the past, most studies reported the asymmetry of strength [24, 37], but this could be connected and influenced by BW distribution, too. Maly et al. [24] reported that more than 73% of soccer players had significant strength asymmetries between legs in both knee flexors and extensors muscle groups. Strength asymmetry is known to decrease with increasing age at professional sport training [37]. However, in our sample, the exact player´s age at professional sport training was not investigated. This could be the reason why we found only nonsignificant changes in BW distribution.

Despite the maturation of the medial longitudinal foot arch during childhood period (4–10 years) [34], some changes in foot morphology may occur during whole lifetime and depend on the physical activity, BW [33], and health conditions of the individual; furthermore, acquired flatfoot type may also develop anytime during one’s lifetime [38]. This is apparent in our longitudinal observation of the FT of elite male soccer players. We observed significant changes in medial longitudinal foot arch morphology during 3 consecutive years in this group of young soccer players, which was represented by the changing distribution of FTs. This could be caused by a combination of many internal and external factors. Players are exposed to a major sporting performance; in that manner, their soft tissues and ligaments could be overused by the long-term term high level performance with not optimal foot morphology and function. Over the course of this study the flatfoot type showed increasing distribution in the sample. One of the considerable reasons could be a summation of the soccer-specific factors. For example, rigid shoes with restricted place for toes, which do not allow a proper stimulation of the interossei muscles to do quality midfoot rocker and relay the impulse to the knee extensors to absorb gravity and ground reaction forces. In that case, the impulse is neither absorbed nor turned to elastic kinetic energy optimally. This may cause the lowering of energy efficiency during walking and running. And with further increasing load of soft tissues and ligaments, the whole process may even get worse resulting in a poor joint alignment with instability [39]. The flatfoot type was found in 17% of the feet: accounting for 11% of the mild form, 3% of the moderate form, and 3% of the severe form, in the first measurement (T1). In the second measurement (T2), the prevalence of the flatfoot type increased to 20%: accounting for 9% of the mild form, 3% of the moderate form, and 2% of the severe form. Finally, in the last year measurement (T3), the total number of flatfoot type was 36%: representing 15% of the mild form, 9% of the moderate form, and 1% of the severe form. While a decrease in the prevalence of the severe form and increase in the prevalence of the mild form of flatfoot type are obvious in our research sample during the entire period, the whole flat foot type group distribution increased simultaneously. On the one hand, this could be explained by the process of maturation when the severe form of flatfoot type could improve during adolescence. On the other hand, the increasing distribution of mild form of flatfoot and finally of whole flatfoot group in our study supports the previously mentioned evidence that the flatfoot type may occur anytime during a lifetime [38] and summation of long-term and repetitive high loading may play important roles. In this regard, we depicted the significant one-leg stance PS changes between measurements T1 and T2. In the NL, we observed worse TTW values than PL values. The lower limbs TTW difference significantly differed only between the first (T1) and third (T3) year of measurement, with constant decreasing of mean value. This improvement of the PS asymmetry could be explained by the improving of lower limbs strength asymmetry with the total age at a professional sport training [37]. It has also been reported that senior professional male players had significantly better PS performance than junior (21 years old) and youth (16 years old) players [40]. This evidence was also supported by the study that compared PS based on different competition levels. This study showed that nationally ranked soccer players had lower PS parameters than regionally ranked players [41]. Hence, the higher the age at professional sport training and the competition level are, the greater the improvement of PS and strength asymmetry is. Interestingly, an excellent correlation was found between the flatfoot type and the PS in young healthy people [17]. As the degree of the flatfoot type increases (the CSI value, respectively) the degree of the PS decreases (the TTW increases, respectively). However, our results showed opposite tendency expressed by the increasing PS degree (decreasing of TTW, respectively) with the decreasing of flatfoot degree during the 3-years of the observation. This supports our hypothesis about the negative impact of longitudinal high loading sport activity on the foot morphology and function. This is in accordance with study of Haendlmayer and Harris [38], in which soccer was indicated to cause prolonged stress and defective biomechanisms on the foot morphology and function possible leading to acquired adult flatfoot type. Regarding to our results, trainers and sport health care providers should check and consider carefully the foot type in adolescence elite soccer players to minimize a risk of musculoskeletal dysfunctions and injury occurrence.

However, the future research is necessary to identify these factors influencing the sport performance and injury risks in adolescent soccer players more deeply with reflection to lower limbs laterality, age at professional sport training, strength asymmetry, and possible correlations between these parameters.

## Limitations

Several limitations need to be stated. This study did not take in account the age at professional sport training of the players and the playing position on the pitch. Additionally, generalizability is another limitation. The study participants consist of elite male soccer players. Thus, the current study results may not be applicable to non-elite and female soccer players.

## Conclusions

We observed changes in foot typology, BW distribution, and PS in young elite male soccer players during 3 consecutive years. We found that lower limbs difference in BW distribution tended to improve during the adolescence; however, statistically significant differences were not detected. We found significant changes in foot typology. There was an evidence of constant increasing in flatfoot type distribution during the study period. In addition, the PS parameter, TTW of COP, showed a significant improvement by the decreasing value in both one-leg stance tests and in the TTW difference between the legs. These results indicated that selected PS parameters and BW distribution could improve with aging. However, a caution needs to be made for an interpretation. Foot morphology and function should be carefully monitored to avoid further problems related with overuse injury in professional young soccer players during maturation. Further research is needed to determine more clear association between these parameters, soccer-related injuries, and sport performances.

## Supporting information

S1 Table (XLSX)
